# Astaxanthin in Exercise Metabolism, Performance and Recovery: A Review

**DOI:** 10.3389/fnut.2017.00076

**Published:** 2018-01-18

**Authors:** Daniel R. Brown, Lewis A. Gough, Sanjoy K. Deb, S. Andy Sparks, Lars R. McNaughton

**Affiliations:** ^1^Sports Nutrition and Performance Research Group, Department of Sport and Physical Activity, Edge Hill University, Ormskirk, United Kingdom; ^2^Faculty of Health Science, Department of Sport and Movement Studies, University of Johannesburg, Johannesburg, South Africa

**Keywords:** antioxidants, carotenoids, *Haematococcus pluvialis*, oxidative stress, inflammation, delayed onset muscle soreness, fat oxidation, endurance exercise

## Abstract

During periods of heavy exercise training and competition, lipid, protein, and nucleic molecules can become damaged due to an overproduction of reactive oxygen and nitrogen species (RONS) within the exercising organism. As antioxidants can prevent and delay cellular oxidative damage through removing, deactivating, and preventing the formation of RONS, supplementation with exogenous antioxidant compounds has become a commercialized nutritional strategy commonly adopted by recreationally active individuals and athletes. The following review is written as a critical appraisal of the current literature surrounding astaxanthin and its potential application as a dietary supplement in exercising humans. Astaxanthin is a lipid-soluble antioxidant carotenoid available to supplement through the intake of *Haematococcus pluvialis*-derived antioxidant products. Based upon *in vitro* and *in vivo* research conducted in mice exercise models, evidence would suggest that astaxanthin supplementation could potentially improve indices of exercise metabolism, performance, and recovery because of its potent antioxidant capacity. In exercising humans, however, these observations have yet to be consistently realized, with equivocal data reported. Implicated, in part, by the scarcity of well-controlled, scientifically rigorous research, future investigation is necessary to enable a more robust conclusion in regard to the efficacy of astaxanthin supplementation and its potential role in substrate utilization, endurance performance, and acute recovery in exercising humans.

## Introduction

Reactive oxygen and nitrogen species (RONS) produced during exercise are recognized as fundamental stressors that can promote improvements in athletic performance and overall health (1–3). To ensure that improvements are sustained, recreationally active individuals and athletes are required to manipulate the volume, intensity and frequency of each exercise bout over time (1). During periods of heavy exercise training, a vast system of interrelated endogenous antioxidant compounds (i.e., superoxide dismutase, catalase, glutathione peroxidase) work together to ensure RONS production does not become detrimental (4). Multifaceted in its function, this system can prevent and delay the oxidation of biomolecules through removing, deactivating and preventing the formation of RONS (5, 6). If exercise training becomes too vigorous, however, an excessive production of RONS can overwhelm the endogenous antioxidant defense system, causing a state of oxidative stress (7). Consequently, lipid, protein and nucleic molecules may become damaged, with potentially detrimental impacts on normal physiological function (8, 9). The intake/ingestion of exogenous antioxidants has, therefore, become common practice in both recreationally active and athletic populations (9). As essential non-enzymatic molecules, exogenous antioxidants have the potential to supplement the endogenous antioxidant defense system if adequately sourced from the diet (10). An example of a potent antioxidant that can be sourced through either dietary intake or supplementation is the xanthophyll carotenoid, astaxanthin. Previously, astaxanthin has been suggested to enhance exercise metabolism, performance and recovery as a result of its potent antioxidant capacity (11–15). Evidence to support this notion has been predominantly derived from studies using *in vitro* and *in vivo* animal models (11–13, 15). The focus of the following review is to, therefore, critically appraise the current literature surrounding astaxanthin and its potential application as a dietary supplement within exercising humans. Underlying mechanistic factors pertaining to exercise metabolism, performance and recovery will also be considered alongside the identification of present and future areas of research focus.

## Astaxanthin

Astaxanthin (3,3′-dihydroxy-β, β′-carotene-4,4′-dione) is a naturally occurring carotenoid found in marine species, such as microalgae, crustacea, fish and some birds (16, 17). Utilized in aquaculture, astaxanthin provides the characteristic reddish pigment to farm-raised salmon tissue (18). Following the initial work of Kurashige et al. (19) and Miki (20), however, an alternative use for astaxanthin as a potent antioxidant compound in both *in vitro* and *in vivo* systems has been suggested. As it is oxygenated (C_40_H_52_O_4_), astaxanthin is classified as part of the xanthophyll sub-species of the carotenoid family (21), with its potency seemingly underpinned by its structure at a molecular level (16, 17, 20, 22). With a molecular mass of 596.84 g·mol^−1^, astaxanthin contains two β-ionone ring systems within its structure that are linked by a polyene chain and contain the oxygenated keto and hydroxyl moieties (21). The presence of the polyene chain alongside each moiety enables astaxanthin to exert multiple antioxidant functions, namely in the scavenging and quenching of RONS within the phospholipid membrane as well as at the surface (20, 22).

### Sources of Astaxanthin

As the human organism is unable to synthesize astaxanthin naturally, its uptake is dependent upon the dietary intake of foods, such as salmon, lobster, shrimp and crab (21). Whilst current data regarding the dietary intake of astaxanthin are insufficient, it is estimated that more than 70% of salmon production worldwide is farm-raised where astaxanthin is utilized as a feeding additive (21). If it is assumed that all dietary sources of seafood are obtained from aquaculture, it is estimated that the average European adult would consume between 0.8 and 2.0 mg·day^−1^ astaxanthin, with the higher percentile not expected to exceed intakes of 4.1 mg·day^−1^ (23). Alternatively, the intake of astaxanthin is possible through regular dietary supplementation, with a typical intake of 4 mg·day^−1^ astaxanthin recommended across several brands of commercially produced astaxanthin products (24). Existing as three separate stereoisomers [(3S, 3′S), (3R, 3′R), and (3R, 3′S)] (25), the commercial applications of astaxanthin range from daily capsules and soft gels to energy drinks and powders (18). Although the production of astaxanthin for aquacultural purposes can be sourced from a range of synthetic and natural sources, the only form currently commissioned for direct human consumption as a commercial product is the (3S, 3′S)-isomer (21, 25). As a result, recent endeavors have been made to improve the production of astaxanthin from natural sources, such as *Haematococcus pluvialis*, to ensure that demands for human applications can be achieved (18).

### Bioavailability of Astaxanthin

Primitive data are available from research that has quantified the uptake and elimination kinetics of astaxanthin in the plasma *via* high-performance liquid chromatography (26–29). This method of analysis was utilized by Rüfer et al. (29) during the investigation of 28 healthy males over a 4-week period. Prior to study onset, astaxanthin concentrations were quantified as non-detectable, following which two randomized groups (*n* = 14 each group) consumed either 250 g of wild or aquacultured salmon daily, to obtain ~1.25 mg·day^−1^ astaxanthin (5 µg astaxanthin·g^−1^ salmon flesh). Following 6 days of consumption, astaxanthin concentrations reached a plateau of 33.7 ± 16.2 nmol·L^−1^ (wild salmon) and 52.4 ± 16.2 nmol·L^−1^ (aquacultured salmon), respectively, with concentrations reported to not significantly change for the remainder of the protocol (29). It therefore appears that when the intake of astaxanthin is chronic, maximal concentrations can be achieved and maintained within the first week of intake, even when astaxanthin is obtained from different sources. This data, however, is collated from one study only, where astaxanthin was consumed as part of the diet, requiring a daily intake of 250 g salmon (29). Future research should, therefore, aim to clarify the bioavailability of astaxanthin from a variety of different sources, including supplementation, with information regarding the elimination kinetics also of importance to understand how the availability of astaxanthin may diminish over time.

Currently, the European Food Safety Authority (EFSA) advise an acceptable daily intake (ADI) of 0.034 mg·kg^−1^·day^−1^ astaxanthin (2.38 mg·day^−1^ in a 70-kg human) (24, 30). Despite this, pharmacokinetic data are available from studies that have issued acute doses of 40 and 100 mg, respectively (26–28). Following the ingestion of an acute *H. pluvialis*-derived 40 mg capsule, maximal plasma concentrations of 55.2 ± 15.0 µg·L^−1^ astaxanthin were recorded in eight healthy male participants (27). In the same study, uptake was significantly enhanced if astaxanthin was ingested as one of three lipid-based formulations (*n* = 8 each group), with maximal plasma concentrations in the range of 90.1 and 191.5 µg·L^−1^ reported (27). As a result, it is advised that astaxanthin should be consumed alongside the intake of dietary fats to ensure uptake can be optimized (31). In comparison, increased maximal concentrations of 1.3 ± 0.1 mg·L^−1^ and 0.28 ± 0.12 mg·L^−1^ are reported in the plasma following an acute intake of 100 mg astaxanthin (26, 28). Although a high level of variance exists between the two studies, this may be explained by the low sample sizes (*n* = 3 each study), which should be addressed in future research (26, 28).

Additional information is also available regarding the pharmacokinetics of astaxanthin following acute supplementation regimes. Indeed, maximal blood astaxanthin concentration is observed between 8 and 10 h following the intake of 40 mg astaxanthin (*n* = 32) (27), with similar times of 6.7 ± 1.2 h (*n* = 3) and 11.5 h (*n* = 3) also observed following doses of 100 mg (26, 28). Furthermore, a half-life of 15.9 ± 5.3 h is reported following a 40 mg dose (27), with half-lives of 21 ± 11 and 52 ± 40 h reported following the intake of a 100 mg dose (26, 28). It therefore appears that the concentration–time profile of astaxanthin is monophasic following ingestion and can be described as a one-compartment model. Future research should seek to replicate these findings in doses equivalent to those advocated by the EFSA (0.034 mg·kg^−1^), as well as those available in commercial astaxanthin products (4 mg). In doing so, optimal dosing strategies can be developed and subsequently implemented for both chronic and acute methods of administration.

### Mechanism of Action

In comparison to other popular phytochemicals, astaxanthin has previously been reported to possess a significantly greater antioxidant function (19, 20, 32), with its antioxidant activities quantified as 10-fold greater than other carotenoids, such as β-carotene, and 100-fold greater than α-tocopherol (vitamin E) (20). In particular, astaxanthin seemingly holds an affinity for singlet oxygen and peroxyl radical intermediates (20–22, 32). Through a process of energy transfer, for example, astaxanthin is able to quench singlet oxygen, yielding ground state oxygen alongside astaxanthin in a triplet-excited state (21). As a carotenoid, astaxanthin is then able to dissipate this energy by interacting with surrounding solvent, returning back to the ground state structurally intact, ready to participate in further quenching cycles (21, 33). Additionally, astaxanthin is also able to scavenge and thus deactivate peroxyl radical intermediates, a function likely dependent upon the formation of resonance stabilized, carbon-centered radical adducts (21, 33). As such, an ability for astaxanthin to extensively protect lipid-rich structures against peroxidation during periods of oxidative stress has been suggested (20, 22, 34, 35).

Research utilizing animal models have also identified a potential for astaxanthin to indirectly modulate the endogenous antioxidant defense system through interacting with redox sensitive transcription factors, such as nuclear factor erythroid 2-related factor 2 (Nrf2) (36, 37). At rest, Nrf2 is sequestered in the cytosol by Kelch-like ECH-associated protein 1 (Keap1); however, during periods of oxidative stress, modifications to cysteine residues on Keap1 cause Nrf2 to be released and translocated to nucleus where it binds to the antioxidant response element (ARE) (38, 39). Once activated, the Nrf2–ARE signaling pathway initiates the transcription of several cytoprotective genes and enzymes capable of upregulating the endogenous antioxidant response to an oxidative stressor, potentially implicating Nrf2 in the beneficial effects of exercise (39). Similarly, phytochemicals can also stimulate the activation of the Nrf2–ARE pathway, a process which may occur through the modification of different cysteine residues to those targeted through exercise, suggesting a potential synergism between exercise and phytochemicals in the upregulation of antioxidant defense (39). Although a specific mechanism of action has yet to be elucidated, research conducted in animal models report increases in Nrf2 expression, alongside the upregulation of endogenous antioxidant enzymes, including superoxide dismutase, catalase and glutathione peroxidase, following astaxanthin administration (36, 37, 40). As key components of the endogenous antioxidant defense system (41), it is plausible that astaxanthin could activate endogenous antioxidant defense pathways upon administration. Future research is therefore required to investigate this mechanism further, including the interaction between astaxanthin and Nrf2 in exercising humans.

### Safety of Astaxanthin Supplementation

In 2014, the EFSA Panel on Additives and Products or Substances used in Animal Feed (FEEDAP) advocated an ADI of 0.034 mg·kg^−1^·day^−1^ astaxanthin (2.38 mg·day^−1^ in a 70 kg human) based upon research previously conducted in rats (30). This was later reiterated by an EFSA Panel on Dietetic Products, Nutrition and Allergies (NDA), where it was concluded that the safety of 4 mg·day^−1^ astaxanthin (~0.06 mg·kg^−1^·day^−1^) had yet to be fully established (24). In contrast, no adverse effects were reported in blood pressure or biochemical parameters, including a metabolic panel and cell blood count, in 19 healthy participants supplementing with 6 mg·day^−1^ astaxanthin for 8 weeks (42). Similarly, no adverse effects were also reported in a recent exercise study where 16 trained individuals supplemented with 20 mg·day^−1^ astaxanthin for 4 weeks (43). The acute intake of 40 mg astaxanthin has also been reported as “well tolerated” in 32 healthy participants with only three mild events in the form of headaches reported in the 48 h post-intake (27). This was, however, in response to a single dose, with information concerning the chronic intake of 40 mg·day^−1^ not currently available in a healthy adult cohort. Nevertheless, the chronic intake of 16 mg·day^−1^ and 40 mg·day^−1^ astaxanthin has been suggested as safe in patients suffering with functional dyspepsia (44). One hundred and 31 patients were recruited and randomly assigned either 16 mg·day^−1^ astaxanthin (*n* = 43), 40 mg·day^−1^ astaxanthin (*n* = 44), or an appearance-matched control (*n* = 44) for 4 weeks. Although 36 adverse events were reported during supplementation and a 4 week follow-up, prevalence was not significantly different between groups (16 mg·day^−1^: 15 adverse events in 10 patients vs. 40 mg·day^−1^: 8 adverse events in 7 patients vs. control: 13 adverse events in 7 patients) suggesting safety (44). As a result, it may be possible to advocate both acute and chronic intakes of astaxanthin that are considerably greater than the current ADI (~2.38 mg·day^−1^). Future research is therefore required to further elucidate the safety of astaxanthin so that human consumption guidelines can be adjusted accordingly.

## Astaxanthin and Exercise Metabolism

The metabolism of fat as an energy source is dependent upon the entry of long-chain fatty acids into the mitochondria; a process requiring the mitochondrial carnitine palmitoyltransferase (CPT) complex and in particular the CPT1 regulatory enzyme (45). During exercise, RONS-induced oxidative damage to CPT1 can alter its function, attenuating the transportation of long-chain fatty acids and consequently limiting the ability for fats to be oxidized as a viable energy source (13). Due to its lipophilic properties, astaxanthin is known to accumulate in the mitochondrial membrane following consumption and provide a protection against RONS-induced detriments to its function (46, 47). It has, therefore, been hypothesized that through its function as an antioxidant, astaxanthin could protect CPT1 against RONS-induced oxidative modifications, causing an indirect enhancement of exercising fat metabolism in the process (13).

Aoi et al. (13) investigated this hypothesis with the use of an exercising mouse-model. In comparison to the control, 4 weeks of astaxanthin supplementation (0.02% w·w^−1^ per 100 g of total dietary intake) enhanced the coimmunoprecipitation of fatty acid translocase (FAT)/CD36 and CPT1 by 14.5% (*p* < 0.05) due to a concomitant 41.4% decrease in the oxidative modification of CPT1 (*p* < 0.05). Muscle glycogen concentrations were also significantly greater in the astaxanthin group (2.7 ± 0.1 mg·g^−1^ tissue) in comparison to the control (2.3 ± 0.1 mg·g^−1^ tissue) and plasma non-esterified fatty acid (NEFA) concentrations tended to be higher (astaxanthin group: 1.05 ± 0.05 mEq·L^−1^ vs. control group: 0.94 ± 0.05 mEq·L^−1^), suggesting a potential glycogen sparing effect (13). In a separate cohort of mice undertaking the same supplementation protocol, the respiratory exchange ratio (RER) was significantly lower from 20 min onward during a 60 min run (25 m·min^−1^), with corresponding measures of fat oxidation (mg·kg^−1^·min^−1^) also 21% higher (*p* < 0.05) and carbohydrate oxidation (mg·kg^−1^·min^−1^) 12% lower (*p* < 0.05) in comparison to the control (13). As a result, it was concluded that the antioxidant potential of astaxanthin is able to indirectly enhance the utilization of fats during exercise through increasing the intercalation of FAT/CD36 and CPT1 on the mitochondrial membrane (13). Similar findings are also reported in a swimming mouse-model (12). On completion of a 15 min swim against an additional 5% body mass, mice supplemented with 6 and 30 mg·kg^−1^ astaxanthin for 3 weeks had significantly greater plasma glucose concentrations (6 mg·kg^−1^: 124 mg·dL^−1^ vs. 30 mg·kg^−1^: 135 mg·dL^−1^ vs. control: 104 mg·dL^−1^), with plasma NEFA also significantly elevated throughout exercise in the 30 mg·kg^−1^ astaxanthin group. Muscle glycogen concentrations were also significantly greater post-exercise following 5 weeks of supplementation with 6 and 30 mg·kg^−1^ astaxanthin (6 mg·kg^−1^ group: 4.0 mg·g^−1^ vs. 30 mg·kg^−1^ group: 4.2 mg·g^−1^ vs. control group: 3.4 mg·g^−1^), with liver glycogen concentration also significantly greater in the 30 mg·kg^−1^ group (30 mg·kg^−1^: 49.8 mg·g^−1^ vs. control: 40.7 mg·g^−1^). In contrast, however, a significant decrement in plasma NEFA concentration has been reported post-exercise (30 min run at 25 m·min^−1^) in mice supplemented with 0.02 w·w^−1^ astaxanthin for 2 weeks (astaxanthin group: 905 ± 41 mEq·L^−1^ vs. control group: 1,151 ± 61 mEq·L^−1^) (15). Although the authors speculated that this resulted from an increased utilization within the skeletal muscle, no direct measurement was made (15). Furthermore, despite a tendency for plasma glucose to be higher post-exercise in the astaxanthin group (astaxanthin group: 121 ± 2 mg·dL^−1^ vs. control group: 112 ± 3 mg·dL^−1^), a significant supplement effect was not reported, suggesting that astaxanthin did not exert a glycogen-sparing effect (15).

Evidence from exercising mice models would therefore suggest that 3–5 weeks of astaxanthin supplementation potentially exerts a metabolic benefit through enhancing fat utilization and attenuating muscle glycogen depletion during endurance-type exercise (12, 13). It should, however, be noted that outcomes reported in an animal model are not always replicable in a human model (48). Interestingly, a similar metabolic effect has yet to be replicated within an exercising human cohort (14, 43). Four weeks of 4 mg·day^−1^ astaxanthin supplementation, for example, did not influence parameters of substrate metabolism (such as RER, carbohydrate and fat oxidation rates, and plasma glucose and NEFA concentrations) obtained during 2 h of submaximal cycling (5% below lactate threshold) in amateur endurance-trained males (*n* = 14; $\dot{\text{V}}$O_2max_ ≥50 mL·kg^−1^·min^−1^) (14). The 4 mg·day^−1^ dose issued, however, was trivial in comparison to the 6–30 mg·kg^−1^ doses that have been previously prescribed in a mouse-model (12). To elicit a similar metabolic modulation as previously reported in a mouse-model, it was therefore hypothesized that a dose greater than 4 mg·day^−1^ would be required in an exercising human cohort (43). As a result, Res et al. (43) recruited trained male cyclists or triathletes ($\dot{\text{V}}$O_2peak_: 60 ± 1 mL·kg^−1^·min^−1^) to a 4 week supplementation protocol consisting of either 20 mg·day^−1^ astaxanthin, or an appearance-matched control in a double-blind, parallel design (*n* = 16 astaxanthin group; *n* = 15 control group). Despite a fivefold increase in dose, however, measures of RER, carbohydrate and fat oxidation rates, and concentrations of plasma glucose and free fatty acids were again not significantly affected during the completion of a 1 h steady-state cycle (50% W_max_) (43). Based upon this evidence, it would therefore appear that 4 weeks of astaxanthin supplementation does not exert a metabolic effect during endurance exercise, even at greater levels of intake, in exercising humans.

Res et al. (43) also detected no measurable changes in the plasma concentrations of the lipid peroxide malondialdehyde (MDA) during exercise, suggesting an antioxidant effect of astaxanthin was not present. Although speculative, this observation may provide an insight as to why a metabolic effect was not reported in this study, with previous research suggesting that an indirect metabolic modulation is mediated through a protection of CPT1 from astaxanthin (13). The lack of a direct antioxidant effect and the corresponding metabolic effect, reported by Res et al. (43), may be attributable to either the high training and fitness demographics of the participants recruited ($\dot{\text{V}}$O_2peak_: 60 ± 1 mL·kg^−1^·min^−1^; training frequency: >3 sessions·week^−1^ for ≥2 years) or the length of the supplementation period undertaken within this study (43). Concentrations of MDA, for example, were reported to decrease significantly in sedentary individuals following 20 mg·day^−1^ astaxanthin for both 8 (astaxanthin group: 1.72 ± 0.28 µmol·L^−1^ vs. control group: 2.08 ± 0.12 µmol·L^−1^) and 12 weeks (astaxanthin group: 1.42 ± 0.29 µmol·L^−1^ vs. control group: 2.00 ± 0.24 µmol·L^−1^), respectively (49). Likewise, 12 weeks of 8 mg·day^−1^ astaxanthin supplementation was also reported to attenuate markers of lipid peroxidation in healthy untrained males (50), suggesting that the demographics of participants may be a confounding variable for the antioxidant action of astaxanthin. Furthermore, the rate at which fat is oxidized during exercise is also subject to large inter-individual variations, even at the same absolute and relative intensities (51–53). The exercise intensity at which fat oxidation is maximal (FAT_max_) is again highly variable between individuals (51, 53). In 300 healthy men and women, for example, FAT_max_ values in the range of 25 and 77% $\dot{\text{V}}$O_2max_ were reported despite an average value of 48 ± 1% $\dot{\text{V}}$O_2max_ reported overall (53). Consequently, if fat oxidation is measured at a constant steady-state intensity, the ability to determine the true effect of a nutritional intervention may be diminished as some individuals will be exercising above and others below the exercise intensity at which FAT_max_ is elicited. Future research should consider participant demographics, such as training status, the supplementation protocols administered, as well as incorporating sensitive measurements of exercising substrate utilization. This may include employing an experimental protocol capable of detecting changes in metabolism across a range of exercise intensities (52, 53), so that the metabolic effect of astaxanthin can be explored in more depth in exercising humans.

## Astaxanthin and Exercise Performance

During endurance exercise, the depletion of muscle glycogen is commonly reported in the etiology of fatigue; as such, methods aimed at attenuating this depletion, may provide an ergogenic benefit through delaying fatigue onset (54). A metabolic mechanism that could potentially convey this benefit is the utilization of fat as an alternative energy source to glycogen during exercise (45). With previous research conducted in mice illustrating such a metabolic effect (12, 13), a potential for astaxanthin to act as an ergogenic aid during the performance of endurance exercise has been hypothesized (12–14, 43).

Ikeuchi et al. (12) conducted a series of experiments in mice to investigate the ergogenic potential of astaxanthin (1.2, 6, or 30 mg·kg^−1^) on swim time to exhaustion (TTE). In the first experiment, mice undertook a weekly swim TTE against an additional 10% body mass over a 5 week supplementation period. In comparison to the control, a continuous significant improvement in TTE was observed from the first week onward in the mice supplemented with 6 (*p* < 0.01) and 30 mg·kg^−1^ (*p* < 0.05) astaxanthin. After 5 weeks, this significant improvement was observed in a dose-dependent manner across all astaxanthin groups with an ergogenic benefit also reported in the 1.2 mg·kg^−1^ astaxanthin group (1.2 mg·kg^−1^ group: 2.27 min vs. 6 mg·kg^−1^ group: 3.32 min vs. 30 mg·kg^−1^ group: 5.12 min vs. control group: 1.44 min) (12). Similar results were also reported in a separate cohort of mice, as 3 weeks of 6 and 30 mg·kg^−1^ astaxanthin supplementation significantly improved swim TTE against an additional 5% body mass (6 mg·kg^−1^ group: 27.50 ± 3.04 min vs. 30 mg·kg^−1^ group: 36.06 ± 4.13 min vs. control group: 19.45 ± 2.02 min) (12). Further support is also evident from a running mouse-model, as mice fed daily with 0.02 w·w^−1^ astaxanthin for 4 weeks were able to significantly enhance TTE at a running intensity of 30 m·min^−1^ by 34% (67.53 ± 4.20 min) in comparison to the exercising control (50.40 ± 5.00 min) (13).

Based upon *in vivo* work conducted in mice, it therefore appears that astaxanthin is able to exert an ergogenic benefit during the performance of endurance exercise; an ability attributed to its aforementioned effect on substrate metabolism (12, 13). In humans, a similar ergogenic benefit was reported in amateur trained male cyclists (*n* = 14; $\dot{\text{V}}$O_2max_ > 50 mL·kg^−1^·min^−1^; weekly cycling volume > 160 km·week^−1^), as 4 weeks of astaxanthin supplementation (4 mg·day^−1^) significantly improved 20 km cycling time trial (TT) performance when compared to baseline (baseline: 2,387 ± 206 s vs. 4 weeks: 2,266 ± 190 s) (14). Furthermore, the 121 s time improvement (5%) reported in the astaxanthin group was also significantly greater than the corresponding 18 s improvement (<1%) reported in the control (baseline: 2,251 ± 260 s vs. 4 weeks: 2,233 ± 283 s), suggesting a treatment effect was present (*p* < 0.05; effect size: 1.25) (14). In contrast, an ergogenic benefit was not reported during a 1 h cycling TT in well-trained male cyclists or triathletes (*n* = 31; $\dot{\text{V}}$O_2peak_: 60 ± 1 mL·kg^−1^·min^−1^; training frequency: >3 sessions·week^−1^ for >2 years) following a 4 week supplementation with either 20 mg·day^−1^ astaxanthin (baseline: 60.38 ± 1.20 min vs. 4 weeks: 59.14 ± 1.21 min) or an appearance-matched control (baseline: 59.43 ± 1.37 min vs. 4 weeks: 58.57 ± 1.39 min) (43). Although there is no clear explanation for the disparity between the two findings, it should be noted that a metabolic mechanism was not reported by either study (14, 43). As the enhancement of fat metabolism and the subsequent sparing of muscle glycogen are the suggested mechanisms by which astaxanthin exerts its ergogenic potential, the absence of this effect may implicate an alternative ergogenic mechanism in Earnest et al. (14), or partially explain the absence of a performance effect in Res et al. (43). It is plausible, however, that the heterogeneity of the sample in Earnest et al. (14) may have contributed to the disparity reported between studies with regards to TT performance. Participants were randomly assigned to each treatment group and although a full familiarization was completed and baseline fitness characteristics appeared to be similar, the SD of each 20 km TT (190–283 s) was far greater than the 136 s time improvement reported in the astaxanthin group. To ensure the reliability of the performance outcome reported, the retest reliability of the 20 km TT should have been reported. As this information is not available, it is difficult to conclude whether the time improvement reported in the astaxanthin group (5%) resulted from a significant supplement effect or the variation present in the study protocol. Previously, a coefficient of variation (CV) of 1.5% (1.1–2.1%; ±10% confidence limits) has been reported for the performance of a 20 km TT separated by 4 weeks in 19 competitive level cyclists (>2 years racing experience at an A or B grade standard) (55). If a similar retest reliability would have been reported by Earnest et al. (14), the ergogenic effect of astaxanthin could have been inferred as the performance improvement during the 20 km TT would have been greater than the CV reported. Future research should, therefore, seek to confirm whether an ergogenic potential of astaxanthin is present within an exercising human cohort, with a particular emphasis placed upon the reliability of testing protocols to ensure that a more robust conclusion can be made. In comparison to previous performance studies, future research should also consider implementing performance tests of a longer duration (>1 h), allowing the ergogenic potential of astaxanthin to be investigated during the completion of an exercise bout that may reflect the proposed mechanism of action more appropriately.

## Astaxanthin and Exercise Recovery

The completion of vigorous intensity exercise training sessions and competitive events are known to increase numerous physiological stressors, such as muscle damage, oxidative stress and inflammation (56). Detriments to the skeletal muscle observed in response to the completion of vigorous intensity exercise may, therefore, not only result from damage directly induced by RONS but also damage induced through the inflammatory cascade. If recovery is inadequate following exercise, it may prevent recreationally active individuals and athletes from completing the subsequent exercise training sessions required to drive adaptation and/or performance improvements. Inadequate recovery may also increase risks of injury, illness and overtraining (57). As a result, the investigation of strategies that can reduce the negative effect of exercise-induced muscle damage and/or accelerate the recovery process has become increasingly popular (56, 58–60).

Through its potency as an antioxidant compound, it is plausible to propose that astaxanthin could exert a recovery benefit through the inhibition of both pro-oxidant and pro-inflammatory intermediates (11, 61). *In vitro* research has reported a dose-dependent inhibition of nitric oxide (NO) and intracellular RONS, as well as the hydrogen peroxide-induced activation of nuclear factor kappa B (NFκB) following 5–100 µM astaxanthin administration (61). Furthermore, 50 µM astaxanthin also completely inhibited the stimulation of inducible nitric oxide synthase (iNOS) and iNOS mRNA. As iNOS is predominantly present in many inflammatory conditions (62), the subsequent expression of pro-inflammatory cytokines, such as tumor necrosis factor-α (TNF-α) and interleukin-1β (IL-1β), were also significantly suppressed (TNF-α: −76%; IL-1β: −46%) in comparison to the control (61). Investigation with *in vivo* mouse models also reported a similar effect following a lipopolysaccharide stressor, with significant reductions in plasma NO (−58%), TNF-α (−50%), and IL-1β (−57%) reported following treatment with a 40 mg·kg^−1^ dose of astaxanthin in comparison to the control (61). Although the work of Lee et al. (61) was not specific to the recovery response to exercise *per se*, it displays a potential mechanism by which astaxanthin may promote recovery through inhibiting the oxidative and inflammatory responses to a physiological stressor. Supportive evidence is, however, received from an exercising mouse model (11). Following a running TTE at an intensity of 28 m·min^−1^, 3 weeks of astaxanthin supplementation (0.02 w·w^−1^) attenuated oxidative damage to lipid and nucleic acid molecules in the skeletal and cardiac muscle tissue. This was evidenced by the substantial decrease of 4-hydroxy-2-nonenal (4-HNE) modified protein production, as well as the significant inhibition of 8-hydroxy-2′-deoxyguanosine (8-OHdG) in the skeletal (−12%) and cardiac (−17%) muscle tissue, in comparison to the control (11). Furthermore, 24 h post-exhaustive exercise, dietary astaxanthin also significantly decreased the activity of myeloperoxidase (MPO) by 50% in the skeletal muscle tissue and 33% in the cardiac muscle tissue in comparison to the control, as well as significantly reducing creatine kinase (CK) activity in the plasma (astaxanthin: 2,617 U·L^−1^ vs. control: 3,032 U·L^−1^) (11). As MPO and CK are markers of the secondary neutrophil infiltration and muscle damage, this also highlights a potential for astaxanthin to attenuate the inflammatory muscle damage response in the days following exhaustive exercise.

The use of *in vitro* and *in vivo* mouse-models have, therefore, demonstrated an ability of astaxanthin to inhibit a series of biomarkers linked to the onset of muscle damage, oxidative stress, and/or inflammation. As a result, research has subsequently aimed to elucidate whether astaxanthin supplementation possesses the ability to modulate the recovery response in an exercising human cohort. In a sample of 20 resistance-trained males, for example, recovery from a muscle damaging bout of eccentric exercise was quantified through perceptions of muscle soreness, concentrations of plasma CK and the recovery of one repetition maximum concentric strength, mean isometric force, and mean dynamic force (63). Although a significant time effect for each measure was present in the 96 h post-exercise, a supplement effect of 4 mg·day^−1^ astaxanthin for 3 weeks prior to exercise was not observed. As young, resistance-trained males were recruited, however, it is uncertain as to whether this outcome could be generalized to less trained individuals or to those participating in events other than high force, resistance exercise. Future research should, therefore, aim to address this uncertainty by recruiting participants with different training demographics to investigate whether training status can confound the efficacy of astaxanthin as a recovery aid post-exercise. More recently, markers of muscle damage and oxidative stress were measured in response to a 2 h bout of soccer exercise in elite youth male soccer players (*n* = 32; age: 17–18 years) following supplementation with 4 mg·day^−1^ astaxanthin for 90 days during the regular competitive season (64). A supplement effect was not reported in exercise-induced changes in plasma CK and generic measures of oxidative stress (thiobarbituric acid-reactive substances, advanced oxidation protein products, superoxide anion, total antioxidant status, sulphydril groups, and superoxide dismutase), suggesting a recovery effect was not present (64). The same research group later conducted a similar investigation in a separate cohort of elite youth male soccer players (*n* = 40; age: 17–18 years) during the competitive season (65). Adherence to exercise training alone over the 90 day protocol was reported to significantly attenuate muscle damage, implicated by a reduction in both plasma CK (astaxanthin: −44.6%; control: −30.1%) and lactate dehydrogenase (LDH) (astaxanthin: −27.4%; control: −18.5%) from baseline measures. Astaxanthin supplementation (4 mg·day^−1^) was suggested to augment these reductions further, while also exerting a secondary anti-inflammatory effect through attenuating training-induced increases in serum C-reactive protein and total leukocyte and neutrophil counts, in comparison to the control (65). Caution is required however, as a significant supplement effect of astaxanthin was not reported in regard to each of the aforementioned measures, apart from the LDH biomarker (*p* < 0.05); suggesting that astaxanthin actually had little effect. Furthermore, when attempting to elucidate the effect of astaxanthin on muscle damage and recovery responses to exercise, both Djordjevic et al. (64) and Baralic et al. (65) placed a heavy reliance upon indirect biomarkers such as CK and/or LDH obtained from the blood. Both CK and LDH have been reported to possess a large inter-individual variance and low reliability in blood concentrations (58), with the use of muscular force production purported to be a more reliable marker of skeletal muscle injury (58, 66). Future research should, therefore, be conducted with the use of suitable analytical methods so that a more comprehensive conclusion can be made regarding astaxanthin and the attenuation of exercise-induced muscle damage in exercising humans.

## Conclusion

Previous research conducted in both *in vitro* cell-cultures and *in vivo* animal models provide evidence to support the use of astaxanthin as a dietary supplement for recreationally active individuals and athletes. According to these models, exercise metabolism, performance and recovery is improved following 3–5 weeks of intake; with each function attributed in part to the potent antioxidant capacity of this xanthophyll carotenoid. In contrast, the current efficacy surrounding astaxanthin supplementation in exercising humans is somewhat equivocal. This is evidenced by the limited number of well-controlled scientific investigations in this research area. Future research is therefore required with a particular emphasis placed upon scientific rigor and contemporary considerations of the most appropriate methodological and analytical strategies. Only once this has been achieved can a more robust conclusion be made about the application of astaxanthin as a dietary supplement for exercising humans.

## Author Contributions

DB, SAS, and LM originally conceived the idea for this review, and DB took overall responsibility for synthesizing the literature and drafting the manuscript. Ongoing critical input was received from LG, SD, SAS, and LM. All authors read and approved the final manuscript.

## Conflict of Interest Statement

SAS and LM have a professional relationship with AstaReal^®^. DB has received external research funding from AstaReal^®^ to undertake postgraduate study. LG and SD have no professional relationship with AstaReal^®^ and have no conflict of interest.
